# Epidemiological pattern of acute respiratory infection among under-fives in Almazar Aljanoubi District -South Jordan

**DOI:** 10.1186/1471-2458-12-S2-A6

**Published:** 2012-11-27

**Authors:** Ibrahim Al-nawaiseh, Ahmad Alkafajei, Jamal Hisham Hashim, Zaleha Md Isa, Nedal Awad Alnawaiseh, Samar Jameel Salahat

**Affiliations:** 1 Jordan German Eye Center, P.O. Box: 13699 Amman 11194 Jordan; 2 Jordan University of Science and Technology, P.O.Box 3030, Irbid 22110, Jordan; 3 Department of Community Health, Universiti Kebangsaan Malaysia Medical Centre, Jalan Yaacob Latiff, 56000 Kuala Lumpur, Malaysia; 4 United Nations University-International Institute for Global Health, Universiti Kebangsaan Malaysia Medical Centre, Jalan Yaacob Latiff, 56000 Kuala Lumpur, Malaysia; 5 Albalqa Applied University, Alkarak College, Alkarak, 61710, Amman, Jordan

## Background

Acute respiratory diseases are the most common international human illnesses. Acute respiratory infection (ARI) in under five year old children represent the most common health problem in paediatric practice all over the world. It is reported that all children under five years suffer from 6-8 respiratory infections annually, which represent about 50% of all preschooler illnesses. The aim of the present study was to assess the epidemiological pattern of ARI and to estimate the prevalence of ARI and the six monthly incidences (person & spells) of ARI among under-five year’s old children in Almazar Aljanoubi district –South Jordan.

## Materials and methods

All children under five years - who attend the Almazar Aljanoubi comprehensive primary health care centre in a sampled three days per week for two successive months from 8am up to 2pm, were enrolled in this study. The mothers of the children were personally interviewed by the investigator. General physical examination was performed. Weight and height were measured.

## Results

Total number of children was 654 (324 males). Negative correlation between age and the prevalence of ARI was observed (Pearson correlation (r) = – 0.832, p < 0.001). The prevalence of ARI was significantly associated with increased overcrowding (t = 6.25, p < 0.001). Rearing of animals and/or birds, and use of kerosene and wood in heating and cooking were significantly associated with prevalence of ARI (OR = 2.2, p = 0.005 and OR = 10, p < 0.001, respectively). Breast feeding was a protecting factor against ARI (OR=2.1, p = 0.048). Bad ventilation of the houses was associated with more episodes of ARI (OR=4.5, p = 0.016). During the period of six months 195 children who develop ARI had 339 episodes among them with a mean duration of 4.5days for each spell (episode).

## Conclusion

Overcrowding was significantly associated with increased prevalence of ARI. Bad ventilation and family history of ARI were significantly associated with more episodes of ARI. Breast feeding were children’s protective factors against ARI.

